# Experimental Analysis on the Effect of Contact Pressure and Activity Level as Influencing Factors in PPG Sensor Performance

**DOI:** 10.3390/s25144477

**Published:** 2025-07-18

**Authors:** Francesco Scardulla, Gloria Cosoli, Cosmina Gnoffo, Luca Antognoli, Francesco Bongiorno, Gianluca Diana, Lorenzo Scalise, Leonardo D’Acquisto, Marco Arnesano

**Affiliations:** 1 Department of Engineering, University of Palermo, Viale delle Scienze, Ed. 8, 90128 Palermo, Italy; francesco.scardulla@unipa.it (F.S.); cosminagnoffo0@gmail.com (C.G.); francesco.bongiorno01@unipa.it (F.B.); gianluca.diana@unipa.it (G.D.); leonardo.dacquisto@unipa.it (L.D.); 2 Department of Theorethical and Applied Sciences, eCampus University, v. Isimbardi 10, 22060 Novedrate, Italy; marco.arnesano@uniecampus.it; 3 Department of Industrial Engineering and Mathematical Sciences, Marche Polytechnic University, v. Brecce Bianche 12, 60131 Ancona, Italy; l.antognoli@staff.univpm.it (L.A.); l.scalise@staff.univpm.it (L.S.)

**Keywords:** photoplethysmography, PPG, PPG sensor, contact pressure, contact force, activity level, influencing parameters, wearables, heart rate, measurement accuracy

## Abstract

Photoplethysmographic (PPG) sensors are small and cheap wearable sensors which open the possibility of monitoring physiological parameters such as heart rate during normal daily routines, ultimately providing valuable information on health status. Despite their potential and distribution within wearable devices, their accuracy is affected by several influencing parameters, such as contact pressure and physical activity. In this study, the effect of contact pressure (i.e., at 20, 60, and 75 mmHg) and intensity of physical activity (i.e., at 3, 6, and 8 km/h) were evaluated on a sample of 25 subjects using both a reference device (i.e., an electrocardiography-based device) and a PPG sensor applied to the skin with controlled contact pressure values. Results showed differing accuracy and precision when measuring the heart rate at different pressure levels, achieving the best performance at a contact pressure of 60 mmHg, with a mean absolute percentage error of between 3.36% and 6.83% depending on the physical activity levels, and a Pearson’s correlation coefficient of between 0.81 and 0.95. Plus, considering the individual optimal contact pressure, measurement uncertainty significantly decreases at any contact pressure, for instance, decreasing from 15 bpm (at 60 mmHg) to 8 bpm when running at a speed of 6 km/h (coverage factor k = 2). These results may constitute useful information for both users and manufacturers to improve the metrological performance of PPG sensors and expand their use in a clinical context.

## 1. Introduction

During the last decades, the persistent improvement in clinical and pharmacological treatments has resulted in a global improvement in the quality of life and life expectancy. Indeed, life expectancy increased by more than six years between 2000 and 2019, as reported by the World Health Organization (WHO) [1]. While on the one hand this represents a great achievement, on the other hand it has led to an increasing number of elderly and frail people requiring healthcare, and people saved from serious acute pathological events but not from the consequent chronic degenerative pathologies [2]. Thus, healthcare providers nowadays operate under unsustainable conditions, which leads to decreased quality and sustainability in the healthcare assistance offered.

Chronic diseases and cognitive impairments pose a crucial burden that requires a change in the healthcare paradigm. Indeed, this is expected to result in new low-cost, high-efficacy interventions and technologies that facilitate new preventive treatments using monitoring systems which prevent rather than cure disease. It is well known that a large proportion of the population could potentially avoid hospitalization or the worsening of their physiological conditions if they were continuously monitored with wearable sensors, not only within a clinical setting but also during normal daily life [3]. Indeed, society wants to age well, pursuing a sustainable lifestyle which enables people to live independently without interference [4], and which also guarantees a personalized comfort level within indoor environments by seeking solutions to support buildings energy efficiency [5]. Furthermore, innovations in both sensing and digital technologies have reached unprecedented levels (partly thanks to artificial intelligence—AI), which can certainly contribute to non-traditional medical approaches which support personalized healthcare that relies (where possible) on wearable, digital, and compact tools that do not require specialized personnel [6].

**Figure 1:**
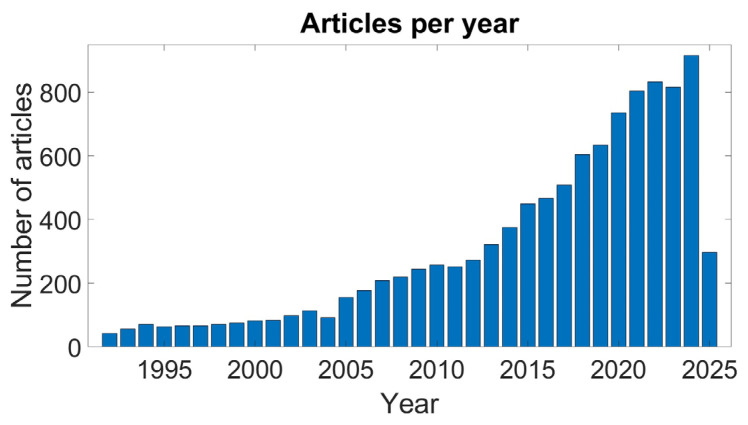
Report on the number of PPG-related articles since 1990. Source: PubMed, April 2025; search query: ((PPG) OR (photoplethysmography) OR (photoplethysmographic)).

In this context, wearable multidomain sensors (e.g., smartwatches and smartbands) can undoubtedly play a pivotal role as they are already adopted and used daily by people who wish to self-monitor their physical activities and their health status [7,8], assess their baseline parameters (e.g., heart rate—HR, respiratory rate, energy expenditure, sleep quality, SpO_2_), and detect any alteration in their normal values. Wearable sensors include a wide variety of monitoring devices which differ in terms of the adopted technology (e.g., optical, piezoelectric, and piezoresistive), the body location (e.g., ears, chest, wrist, and forehead), and the detected parameters. Among the wearable devices, the most widely used technology for the detection of cardiovascular parameters is photoplethysmography (PPG), which is based on an optical sensor and often known simply as a PPG sensor. Figure 1 shows the growing trend of interest in PPG sensors during the last decades within the scientific community.

Their implementation is supported by several aspects: small size, low cost, constructive simplicity, and the potential variety of cardiovascular parameters that can be detected [9,10]. These factors have driven the integration of this technology within various wearable devices of mass consumption, such as smartwatches, making this technology available to a significant portion of the population in developed countries [11].

The principal components of a PPG sensor are (i) a light source (LED), which is usually between 525 and 780 nm, and (ii) a photodetector (PD). These components can be arranged in two different configurations: transmission mode, in which the measuring site is in between the LED and the PD, and reflective mode, in which the LED and the PD are on the same plane with the measuring site underneath. This latter configuration, which is capable of providing a good signal [12], is suitable for integration into wearable devices such as smartwatches.

The PPG sensor can detect blood volume variations: every time the heart beats it pumps blood within the vessels, which in turn increases their volume to accommodate the larger amount of blood. This variation in blood volume is detected by the PPG sensor as a variation on the electrical output of the sensor, which is proportional to the light absorbed by blood. The PPG signal constitutes a direct current (DC) component, which corresponds to all the tissues and does not vary rapidly over time, and an alternating current (AC) component, which corresponds mainly to the blood volume variation that occurs at every heartbeat. Through the analysis of the PPG signal, it is possible to derive multiple parameters of the cardiorespiratory system, comprising HR [9], blood oxygenation [13,14], blood pressure (BP) [15], respiratory rate [16], and arterial stiffness [17], among others. Thus, the PPG sensor has huge potential for monitoring a subject’s health status such as via patient monitoring [10] and daily activities monitoring [18], in the training of athletes [19], and for ensuring workers’ safety [20].

Despite these strengths and its transversal applicability, the amplitude of the AC component represents just a fraction of the whole PPG signal, exposing it to different influencing parameters and limiting its accuracy and clinical usefulness [9], ultimately making it difficult to acquire a reliable PPG signal during daily routine activities and physical exercise. Among the different parameters of influence [9] the most significant are as follows: the contact pressure (CP) [21,22,23], which can be defined as the pressure between the skin and the PPG sensor, the wavelength of the light emitter [24], the skin tone [25], the LED-PD distance and configuration [26], motion artifacts [27], ambient light [28], and temperature [29]. Among these, the CP is reported to have a significant influence on the PPG amplitude and on minimizing the other influencing parameters such as the ambient light and the relative motion between the sensor and the skin during physical activities [9]. Specifically, a reduced CP may cause relative motion between the sensor and the skin which results in noise, and it allows ambient light to further degrade the signal quality. Conversely, an excessively high CP can alter the measurement site microcirculatory conditions because of the compression of the capillary bed at the point where the information is obtained. Thus, it appears even more evident that there is a need for sound testing and validation methodologies, delivering homogeneous and comparable results that robustly qualify a device from a metrological point of view [30].

From the perspective of the broader usability of PPG-based wearable sensors in clinical and (more in general) physiological monitoring settings, it is mandatory to improve their metrological performance; the development of sensor configurations and settings will also enhance the reproducibility of the measurement results generated in wildlife conditions. On another hand, it is fundamental to define rigorous test methodologies and measurement procedures to obtain reproducible results that can be compared with other studies in the literature [31]. This can undoubtedly contribute to a better understanding of the fundamentals of PPG functioning and its influencing parameters [9] and pave the way to a wider implementation of this sensor in clinical settings as well as other application fields. In particular, the present study has been carried out using the framework described in the WEPOP (WEarable PlatfOrm for OptImised Personal comfort) project, aiming at defining a wearable system (also relying on PPG technology) for the assessment of personalized comfort in living environments. The sensors employed allow us to gather data exploitable for the definition of Personal Comfort Models (PCMs) that can predict the actual status of the occupants’ and to improve the conditions from a human-centric view, whilst also enabling an improvement from an energy consumption point of view. It is beyond doubt that the quality of the data collected directly impacts the accuracy of such models and hence influences the final contribution that such a methodology can have to the overall enhancement of the quality of the global ecosystem, considering both the living environment and its dwellers.

Based on these considerations, the future of medicine and personal physiological monitoring in everyday life lies in the uniformity and reliability of the testing procedures and on the metrological quality of measurements. Therefore, it is essential to improve the quality of the PPG sensors through a better understanding of the principal influencing parameters, such as CP. Assessing the optimal contact pressure, which may differ depending on each individual [9,32], is still challenging but may have significant benefits in improving the overall accuracy and usability of the PPG sensors. Hence, the aim of this paper is to perform a metrological characterization of a PPG-based wearable prototype in relation to different CP values (i.e., 20, 60, and 75 mmHg) and diverse walking/running speeds (i.e., 3, 6, and 8 km/h). The tests were carried out on 25 healthy volunteer subjects; it is worth underlining that this is not a clinical study and does not aim to certify any device as a medical product. A cardiac belt (based on electrocardiography, ECG) was employed as a reference sensor (gold standard); it is worth underlining that this metrological characterization does not aim to certify the accuracy of the sensors for clinical purposes nor for marketing, hence a medical-grade gold standard has not been employed in the test campaign.

## 2. Material and Methods

The tests were performed in two different laboratories: one at Università degli Studi di Palermo (Palermo, Italy) and one at Università Politecnica delle Marche (Ancona, Italy). The tests were conducted according to the same test protocol and in compliance with the ethical guidelines of the two universities. Before starting the tests, the aims and methodologies of the study were clearly explained to the participants; they signed an informed consent form highlighting the test protocol and conditions and stating that no risks were present. The participation was voluntary, and no compensation was provided. HR measurements acquired through a PPG sensor with controlled contact pressure and an ECG-based reference system were performed to evaluate how different contact pressure levels affect the PPG signal during different physical activity intensity levels. The general methodology complies with an adopted standard which regulates the procedures and aspects that should be observed during performance tests conducted on PPG sensors. Data analysis reflects the most adopted approaches for this type of study; all the data were managed according to the General Data Protection Regulation (GDPR). In the following subsections, the experimental setup, the experimental protocol, and the data analysis are described in detail.

### 2.1. Experimental Setup and Equipment

**Figure 2:**
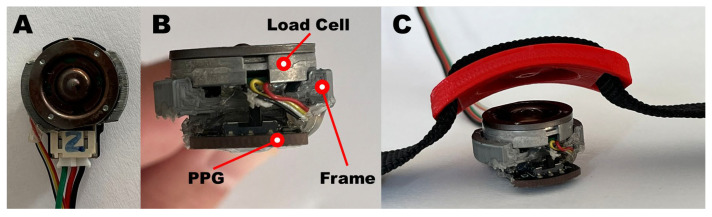
PPG-based device comprised of a PPG sensor and a compression load cell; (**A**) top view, (**B**) front view, and (**C**) integrated with a bracelet.

A PPG sensor equipped with a single LED with a wavelength of 520 nm (DFRobot, Beijing, China) was integrated in a 3D-printed polylactide frame, designed to be worn on the wrist via a bracelet and to host a bottom load cell (FX1901, Meas.Spec., Fremont, CA, USA) stack on the PPG sensor itself, as shown in Figure 2. Such a configuration allows the load cell to provide the desired contact pressure between the PPG sensor and the skin.

Specifically, the printed components are composed of a frame and a cell press (Figure 2B and Figure 2C, respectively). The first component has two main functions: it is designed to act as an interface between the PPG sensor and the load cell and to keep the two surfaces parallel. The second component, which has two slots for the 2 cm nylon bracelet, is used to transmit the pressure exerted by the bracelet to the load cell, facilitated by the matching surface which is specular to that of the load cell. The combination of the two components allows us to normally transmit the force generated by the tightening of the strap directly on the load cell and on the underlying PPG sensor. A ratchet was used as a fastening system for the bracelet so that once the desired contact pressure is reached, the lock system prevents any loosening.

The calibration of the load cell, which directly provides the value of the pressure exerted by the sensor on the skin in mmHg (1 mmHg = 133,322 Pa), was conducted by stacking a series of calibrated masses of 110, 300, and 360 g on the whole load cell.

**Figure 3:**
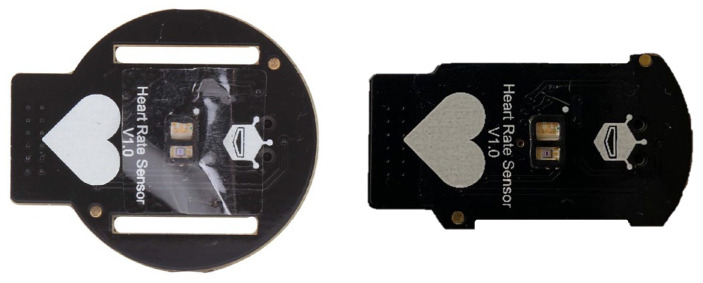
Original PPG sensor surface (on the left) and final (on the right) obtained after the milling process.

During the tests, to determine the higher contact pressure values without causing an uncomfortable feeling of constriction on the participants’ wrists, the printed circuit board on the PPG sensor was milled into the lateral side with no electrical circuits (Figure 3).

This allowed us to reduce the sensor area from approximately 460 mm^2^ to an area of about 355 mm^2^, thus a reduction of 25%.

The signal provided by the load cell was amplified through the integrated HX-711 (SparkFun Electronics, Niwot, CO, USA) and all the components were managed using an Arduino Uno microcontroller at a sampling frequency of 80 Hz.

The HR reference data were acquired via an ECG-based chest strap (H9, Polar Electro, Kempele, Finland) and a mobile app (Elite HRV Inc., Austin, TX, USA) which recorded a text file containing all the R-R intervals from which the HR was finally calculated and synchronized with the PPG-derived HR.

All the physical activities were performed on a treadmill (JK Fitness, Movifitness MF Top Slim, Padua, Italy) at different speeds (i.e., 3 km/h, 6 km/h, and 8 km/h; 1 km/h = 0.278 m/s) with the slope fixed at zero degrees. These values were chosen to reflect walking, jogging, and light running activities; additionally, these speed values are consistent with the ANSI/CTA-2065 recommendations [33].

Blood pressure was registered before every physical activity session through an automatic oscillometric device (Omron, M2, Kyoto, Japan) validated according to the Universal Protocol [34].

### 2.2. Experimental Protocol

The test protocol was designed in accordance with the standard ANSI/CTA-2065 [33], proposed and released in 2018 by the Consumer Technology Association; it aims to define the process necessary to test and validate the accuracy of a device for HR monitoring under different conditions, providing indications on different aspects including the test conditions and the protocol. It also suggests an ECG system as a reference device. Although chest straps are widely used and accepted in the literature [31,35], it is worth mentioning that studies for assessing the measurement accuracy of wrist-worn devices have also adopted 12-lead ECG systems as a reference [36].

Twenty-five subjects (10 males and 15 females; age: 23.9 ± 2.8 years, range 22–36; height: 171 ± 8 cm; weight: 67 ± 11 kg—values are reported as mean ± standard deviation) were enrolled in the study after signing informed and data consent forms. None of the enrolled subjects reported any known cardiovascular diseases nor reported taking any medications that can influence the main parameters of the cardiovascular system. No details on the general medical history or the subjects’ anamnesis were requested from the participants; additionally, no hypertensive or hypotensive subjects were included in the study.

All the tests, which had an overall average duration of approximately 60 min each, were conducted in laboratories with room temperatures ranging from 22 °C to 24 °C.

Before starting the tests, the consensus and the anthropometric information for each subject were collected and managed in compliance with the General Data Protection Regulation in order to preserve the participants’ privacy. The skin color of each participant was determined by the same single operator by comparing each participant’s arm (inner part, where the PPG sensor would come into contact) with the Fitzpatrick chromatic scale displayed on a high-definition screen with a fixed brightness; three subjects presented a skin classification of type 1, nine subjects had a skin classification of type 2, 13 subjects had a skin classification of type 3.

**Figure 4:**
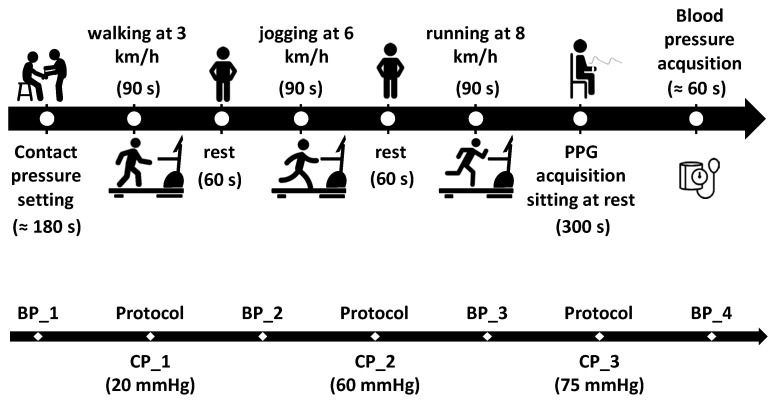
Description of the protocol (top), which is repeated for each contact pressure (i.e., 20, 60 and 75 mmHg) and a summary of the blood pressure acquisitions for each subject (bottom).

At the very beginning of each test, a first blood pressure measurement (BP_1) was recorded. Then, the study coordinator asked each participant to wear the Polar H9 chest strap and then to sit and rest on a chair while placing the prototypal bracelet on the left wrist, ensuring it was tightened to the first contact pressure of 20 mmHg (CP_1). The wires from the bracelet were then fixed onto the arm using adhesive tape to prevent any unwanted displacement of the system during the physical activity execution. Thus, each subject began the planned activity as depicted in Figure 4.

Specifically, each participant was asked to perform three test sessions on a treadmill at different speeds (i.e., 3, 6, and 8 km/h for 90 s at each speed), with 60 s rest in between. After the third session at 8 km/h, each participant was asked to sit still at rest for 5 min while acquiring the PPG signal and then to have the last blood pressure measurement taken (BP_4).

At the end of each session, the coordinator, after verifying that the tightening pressure of the bracelet had not decreased during the physical exercise, tightened the bracelet to the next pressure level (i.e., 60 mmHg (CP_2) and 75 mmHg (CP_3)) and asked the participant to repeat the treadmill session (Figure 4).

### 2.3. Data Analysis

The collected ECG and PPG data were post-processed in the MATLAB^®^ (v. 2024b) environment. At first, the digitized PPG waveform was filtered with a combination of a fourth order Butterworth IIR band-pass filter (0.5–5.0 Hz) and a Hampel filter in order to remove outliers [37,38]. The Butterworth filter is extensively used in processing PPG signals as it guarantees the least signal distortion [39,40]. Nevertheless, it is worth mentioning that it may have introduced phase distortion to the pulse waveform [41].

HR values were computed from both the ECG (reference) and the PPG (test) signals; for the former, the RR values provided were converted into HR [bpm], whereas for the latter, the signal peaks were detected first, hence the PP intervals (i.e., the inter-beat intervals) were computed and the HR values were derived (using the same procedure adopted for the ECG sensor signals). Hence, the HR values as extracted from the ECG and PPG signals were synchronized by searching for the maximum correlation values (i.e., looking for the minimum difference in terms of beat per minute). Then, the test and reference HR values (the 50 central beats from each acquisition) were compared; the metrics employed to evaluate the metrological performance of the test PPG sensor were as follows: (i) the mean absolute percentage error (MAPE) and (ii) the confidence interval at 95% derived from the Bland–Altman plot. The strength of the linear correlation between the HR values derived from the test and reference devices was assessed using the Pearson’s correlation coefficient (ρ). Also, the measurement differences (i.e., residuals) were analyzed; their mean and standard deviation values can be related to the measurement accuracy (or rather, trueness) and the precision, respectively.

## 3. Results

It is worth underlining that the CP set before starting physical activity changed during the test execution due to the arm movements and the contraction of the muscles. However, at the end of the test, the CP value returned to within ±5 mmHg of the preset value. This means that the tightening system equipped with a ratchet was adequate and did not loosen during the activities execution.

In total, nine of the 225 acquisitions were corrupted or unavailable due to technical issues such as cable disconnection, hence 216 series were finally analyzed.

Table 1 reports the BP acquisitions during the tests, as described in Figure 4, together with the HR values provided by the oscillometric device. The average systolic and diastolic blood pressure measurements were equal to (113 ± 12) mmHg and (75 ± 7) mmHg, respectively (reported as mean ± standard deviation values), with a difference of (3 ± 7) mmHg for the systolic and (1 ± 8) mmHg for the diastolic blood pressure between the first and last measurement from each test. No significant correlation between the results of the tests and the blood pressure was found. Average HR during the BP acquisitions displayed a positive trend, increasing from 73 bpm to 87 bpm. The average HR provided by the ECG-based device was equal to (105 ± 5) bpm at 3 km/h, (120 ± 11) bpm at 6 km/h, and (136 ± 15) bpm at 8 km/h.

**Figure 5:**
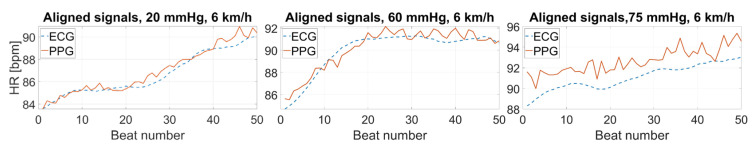
Examples of the test (PPG) and reference (ECG-based) acquired signals (speed: 6 km/h; contact pressure: 20, 60, 75 mmHg; skin color: 2; subject 23).

An example of the synchronized test and reference signals is reported in Figure 5 for the test (PPG sensor) and the reference (ECG-based device) systems.

The accuracy and precision at each speed and for each contact pressure were assessed using the MAPE (Table 2) and the Pearson’s correlation coefficient (Table 3), as well as through an analysis of the deviations between the HR values measured by the PPG-based and the ECG-based systems (Table 4).

**Figure 6:**
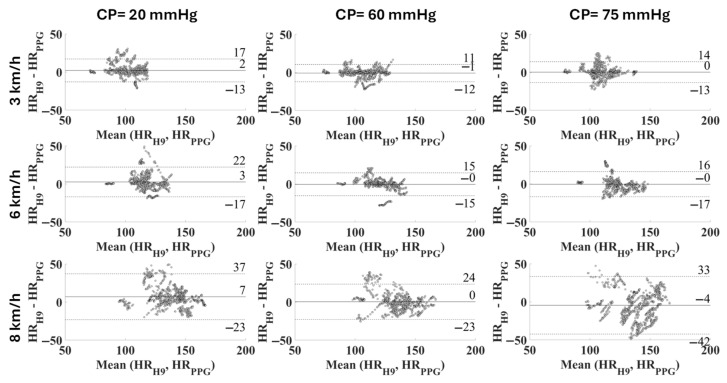
Bland–Altman plot related to each combination of CP and speed in the cohort of subjects (expressed as average difference ± 1.96 standard deviation of the measurement differences).

**Figure 7:**
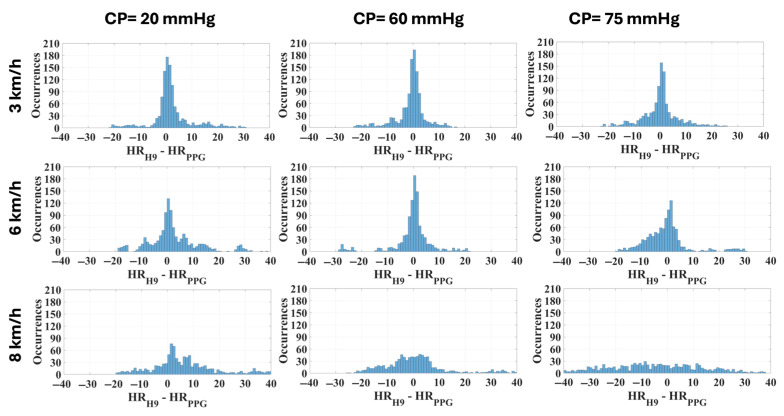
Distribution of deviations related to each combination of CP and speed in the cohort of subjects (width: 1 bpm).

The comparison between the measurement systems was further tested using the Bland–Altman plot with confidence intervals at 95% (Figure 6) and through the distribution of deviations with respect to the reference device (Figure 7).

**Figure 8:**
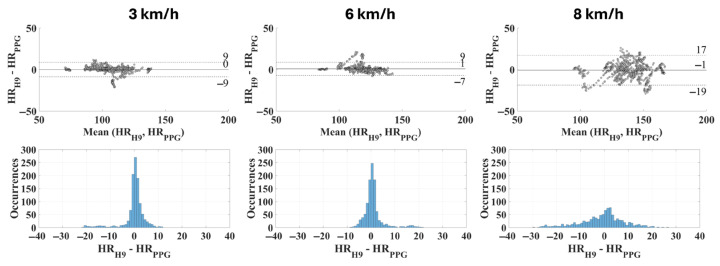
Bland–Altman plots and distribution of deviations related to the optimal contact pressure for each subject (bin width: 1 bpm).

Finally, the most reliable CP (i.e., the one which provides the lowest MAPE) is not a single value for all subjects. Beyond the physical activity level, it also depends on several factors including the specificities of each individual in terms of their vasculature properties; therefore, the optimal individual contact pressure was identified for each subject. Then, the same metrics previously adopted were recalculated; these are presented in Table 5 and in Figure 8.

## 4. Discussion and Conclusions

In this paper the authors evaluated the effect of different contact pressure values on the quality of the data gathered through a wrist-band wearable sensor. In particular, the analysis was performed on the heart rate (HR) values derived from a test (prototypal bracelet) device and a reference (Polar H9 chest strap) device, collecting photoplethysmographic (PPG) and electrocardiographic (ECG) signals, respectively. It is worth mentioning that the ECG-based chest strap, which is the most widely-adopted reference system, does not provide information regarding its accuracy during physical activities; on the other hand, it is beyond doubt that a medical-grade 12-lead ECG device would be the best choice as the gold standard. However, since medical certification is beyond the aim of this work at present, the Polar H9 device can be considered suitable (additionally, it has been extensively employed in the literature as a gold standard instrument [42,43]), and any uncertainty has been assumed to be included in the expanded uncertainty estimated in the analyses.

An extensive metrological evaluation was performed to evaluate the effective performance of the proposed PPG-based prototype in the measurement of the HR during different intensities of physical activity. First of all, it is crucial to underline the adequateness of the tightening system, which allowed the substantial maintenance of the contact pressure during the tests without loosening issues. Plus, limited variations in blood pressure values were observed before and after the dynamic exercises, but no further considerations were explored on the impact of blood pressure and physical activity intensities [44].

Observing the synchronized signal portions (Figure 5), it is immediately evident that the contact pressure directly affects the agreement between the HR values derived from the test and reference devices, leading to increasing measurement differences when the sensor adherence is not adequate and, thus, to a worsening signal quality. Consequently, this leads to a higher measurement uncertainty, which can undermine the reliability of the usage of PPG-based devices during certain exercises. This becomes particularly relevant in sport applications and in the context of wellbeing assessments, which are among the main application fields for wearable sensors [45,46].

The results in terms of the performance metrics (i.e., the mean average percentage error and correlation coefficient—Table 2 and Table 3) show that the intermediate contact pressure (CP), i.e., 60 mmHg, is the optimal choice considering all the activity levels (i.e., walking/running at 3, 6, and 8 km/h). In fact, this CP value consistently allowed the achievement of the lowest MAPE, with average values ranging from 3.36% to 6.83% depending on the walking speed/activity intensity level, versus 4.99–9.12% for CP = 20 mmHg and 4.37–11.85% for CP = 75 mmHg. This was similar for the correlation coefficient; the CP = 60 mmHg always resulted in a value higher than 0.70 (achieving 0.88 at 3 km/h). This result is in line with the findings of the previous work of some of the authors [21], where a CP of 54 mmHg was identified as optimal, with a MAPE ranging from 2.4% to 3.8% and a Pearson’s correlation coefficient of 0.81–0.95 at different physical activity intensities (corresponding to HR values of 90 bpm—low intensity, 120 bpm—medium intensity, and 140 bpm—high intensity). Such results confirm that the proposed methodology allows us to obtain accurate results, with a MAPE < 5% and a strong linear correlation with the reference device. This cannot be stated for the activity speed of 8 km/h, which on the contrary leads to excessively impactful motion artifacts, impairing the quality of the results.

However, in the previous study [21] no contact pressure values higher than 54 mmHg were analyzed and the different levels were chosen by asking the participants to wear the bracelet as a smartwatch with different tightening levels. Thus, the objective of the present study was to assess the metrological performance of the PPG sensor with pressures higher than 54 mmHg, chosen empirically based on the previous study. Although theoretically the optimal contact pressure should be equal to the transmural pressure [9], a recent study [47] reported that the PPG signal responses may be dependent on the wavelength and on the skin and vasculature properties as the CP changes. Thus, the results, in addition to confirming those previously obtained, demonstrate that 75 mmHg is an excessive pressure that causes a significant decrease in precision at different speeds (Figure 7), especially at 8 km/h.

These considerations are directly reflected in the analysis of the measurement differences (residuals), as observed in Table 4. The distribution is tighter with a CP equal to 60 mmHg and a walking/running speed up to 6 km/h (Figure 7). Precision and trueness reached their best values at the same CP of 60 mmHg for any intensity of physical activity, as seen in Table 4 and Figure 6. Specifically, the confidence intervals (coverage factor k = 1.96) varied from [−12, 10] bpm at 3 km/h to [−23, 23] bpm at 8 km/h, with a mean value of −1 bpm and 0 bpm, respectively. At 8 km/h the residuals seem not to have a defined distribution, appearing almost casual.

Evaluating the effect of the contact pressure on the quality of the PPG signal during activity execution is extremely interesting, since PPG-based wearables are notably prone to motion artifacts. Studies in the literature [20] consider the HR measurement as reliable when the mean absolute percentage error (MAPE) is lower than 5% and the linear correlation with the reference value is strong (i.e., ρ > 0.7). Upon this basis, all the values measured at a walking speed of 3 km/h can be considered reliable (MAPE within the range 3.36–4.99, ρ ≥ 0.77), regardless of the CP (Table 2). On the other hand, when running is considered, the results cannot be considered acceptable at a CP of 20 mmHg (MAPE = 6.11%, ρ = 0.62), but they are acceptable at higher CP values (best with a CP of 60 mmHg, with MAPE = 3.62% and ρ = 0.80, whereas with CP = 75 mmHg MAPE is 4.73% and ρ is 0.75, and this still considered reliable). At 8 km/h none of the CP values allow us to obtain accurate HR values; this can be attributed to the high variability in the measurement results, as can be observed in the Bland–Altman plot (Figure 6, right). If a specific CP value is considered, the MAPE increases with the activity level, as expected, due to possible motion artifacts. On the other hand, the value of the correlation coefficient decreases due to a worsening correlation with the reference values. It is feasible to assume that at the highest contact pressure (i.e., 75 mmHg) and at the highest exercise intensity (i.e., 8 km/h) the effect of the tightening, combined with the lower damping, has a compressive effect on the capillary bed, causing a significant decrease in the signal quality.

However, it is worth mentioning that any given contact pressure is not necessarily the optimal one for a different subject; this is attributable to the intrinsic inter-subject variability found in the wrist morphological characteristics and in the cardiovascular properties. In fact, if individualized optimal values are considered, the measurement uncertainty significantly decreases (Table 5) from 15 bpm (with a CP equal to 60 mmHg) to 8 bpm when considering a running speed of 6 km/h (coverage factor k = 2). At the same time, the accuracy (or rather, trueness) is optimal, with a mean residuals value equal to 1 bpm (instead of 0 bpm when considering the same running speed). This is reflected also in the distribution of the residuals (Figure 8), which appears very high and tight up to 6 km/h (standard deviation equal to 5 bpm and 4 bpm at 3 km/h and 6 km/h, respectively); indeed, at 8 km/h the measurement uncertainty is almost doubled (standard deviation of 9 bpm).

In conclusion, it is evident that the contact between the sensor and the skin is a crucial aspect in PPG-based sensors and a system capable of optimizing the contact pressure depending on individual characteristics would be beneficial and could also widen the applications of such devices. The possibility of personalizing the sensors can improve the measurement accuracy and this could be widely exploited in fields like indoor comfort assessment (also during movements) and sport applications (where motion is unavoidable and needs to be thoroughly considered without impairing the accurate measurement of physiological signals), just as examples. In future, it would be interesting also to extend the study to subjects with different skin tones, hence covering most of the Fitzpatrick scale; it is worth underlining that the skin tone assessment can be performed on different body areas provided that they are protected from sunlight and hence representative of the effective skin phototyping of the subject (e.g., other works in the literature use the inner part of the upper arm) [48].

These results and the considerations that can be inferred are relevant to the fine-tuning of a wearable system capable of accurately measuring the physiological signals that can be exploited to provide an insight into the overall status of the subject, while also reflecting their personal perception of the indoor comfort in a certain living environment. This can contribute to the improvement of the quality of living and in future could be extended also to clinical settings (this would require a clinical research study for validation purposes and marketing of the device, according to national and international guidelines and recommendations, e.g., EU Regulation 536/2014—Clinical Trial Regulation (CTR) [49] and EU Directive 2001/83 [50], among others). Additionally, it is worth noting that the results from such experimental tests can be exploited for the optimization of the hardware design of wearable sensors capable of auto-tightening the bracelet around the wrist, hence improving the contact pressure between the PPG sensor and the skin and hence, the quality of the measured data. In turn, these data can inform PCMs that will be better performing and consequently more effective in the enhancement of indoor well-being, as well as in the building energy consumption through the integration of the monitoring data analysis within the building management and control systems.

## Figures and Tables

**Table 1:** Blood pressure values acquired during the protocol (Figure 4). HR values were provided by the oscillometric device together with the BP measurements and are reported as mean ± standard deviation.

	Systolic BP [mmHg]	Diastolic BP [mmHg]	HR [bpm]
BP_1	114 (12)	74 (7)	73 (12)
BP_2	115 (15)	75 (6)	82 (14)
BP_3	113 (12)	75 (7)	86 (17)
BP_4	111 (11)	74 (7)	87 (16)

**Table 2:** Performance metrics at different speeds at each contact pressure: MAPE at each physical activity level and for each contact pressure.

	MAPE [%] (σ)		
	CP [mmHg]		
Speed [km/h]	20	60	75
3	4.99 (6.38)	3.36 (3.49)	4.37 (3.73)
6	6.11 (6.54)	3.62 (4.59)	4.73 (5.67)
8	9.12 (9.44)	6.83 (6.38)	11.85 (8.56)

**Table 3:** Performance metrics at different speeds at each contact pressure: Pearson’s correlation coefficient of PPG-HR.

	ρ [−]		
	CP [mmHg]		
Speed [km/h]	20	60	75
3	0.77	0.88	0.83
6	0.62	0.80	0.75
8	0.60	0.75	0.44

**Table 4:** Analysis of residuals: mean and standard deviation values, linked to measurement accuracy and precision, respectively.

	Residuals μ ± σ [bpm]		
	CP [mmHg]		
Speed [km/h]	20	60	75
3	2 ± 7	0 ± 5	0 ± 6
6	2 ± 9	0 ± 7	0 ± 8
8	6 ± 15	0 ± 11	−4 ± 19

**Table 5:** Performance metrics at different speeds using the optimal CP for each subject: MAPE, Pearson’s correlation coefficient, and residuals analysis.

	Optimal Contact Pressure (mmHg)		
Speed [km/h]	MAPE [%] (σ)	ρ [−]	Residuals μ ± σ [bpm]
3	2.38 ± 2.99	0.94	0 ± 5
6	2.07 ± 2.58	0.93	1 ± 4
8	4.90 ± 3.09	0.83	−1 ± 9

## Data Availability

Data will be made available upon request to the corresponding Author.
